# Longer-term impacts of flooding on Australian health systems: insights from medical students on rural clinical placement

**DOI:** 10.1186/s12913-026-14171-4

**Published:** 2026-02-19

**Authors:** Jodie Bailie, Karen Scott, Christine Ahern, Joseph Duncan, Rebecca McNaught, Ross Bailie

**Affiliations:** 1 University Centre for Rural Health and Centre for Disability Research and Policy, The University of Sydney, 61 Uralba Street, Lismore, NSW 2480 Australia; 2 Sydney Medical School, The University of Sydney, Anderson Stuart Building, Camperdown, NSW 2050 Australia; 3 University Centre for Rural Health, The University of Sydney, 61 Uralba Street, Lismore, NSW 2480 Australia; 4 School of Public Health and The University Centre for Rural Health, The University of Sydney, 61 Uralba Street, Lismore, NSW 2480 Australia

**Keywords:** Climate change, Disaster, Flood, Health system, Health service, Medical education

## Abstract

**Objectives:**

To explore the perspectives of medical students in diverse clinical settings to enhance understanding of the sustained, system-wide effects of flooding on health systems.

**Design:**

A qualitative descriptive study using interviews and focus groups (17 April − 12 May 2023). Data were first analysed using inductive content analysis, and an analytical framework was then developed through an iterative process. The analysis and interpretation were refined with input from co-authors, including clinicians based in the region during and after the flooding events.

**Setting:**

Rural area of the Northern Rivers region, New South Wales, Australia, 14 months after major river flooding in 2022.

**Participants:**

Medical students undertaking rural clinical placements who were in the region after the flooding event.

**Results:**

Of the 39 students in the cohort, 32 (82%) participated. Four key domains of ongoing impact were identified: community, health services, clinicians, and broader social and physical environments. Ten categories emerged, highlighting the breadth of ongoing impacts. These included community psychological trauma, disruptions to health services, staffing challenges, and delays in patient discharge. Significant psychological impacts on healthcare workers were observed, with burnout affecting the delivery of medical education. Displacement was found to limit both access to healthcare and broader health outcomes. Additionally, the floods disproportionately affected socially vulnerable groups, and delays in government housing support contributed to ongoing stress within affected communities.

**Conclusion:**

Our findings demonstrated that catastrophic flooding disrupted health service delivery and community wellbeing, with impacts including psychological trauma, workforce strain, displacement, and disproportionate effects on socially vulnerable groups, as well as consequences for medical education. They highlight the importance of supporting medical students to reflect on and learn from these experiences. The analytical framework developed, grounded in student perspectives and consistent with clinician experiences, contributes to the foundation for future research, intervention design, and preparedness planning.

**Supplementary Information:**

The online version contains supplementary material available at 10.1186/s12913-026-14171-4.

## Introduction

Given the escalating frequency and severity of disasters due to climate change, it is crucial to understand their impacts on health systems, including the health workforce, and to prepare accordingly [1–4]. Globally, floods are the most frequent type of weather-related disasters [5], placing considerable strain on health systems by disrupting the delivery and accessibility of healthcare to affected populations. In their immediate aftermath, such events can lead to damaged infrastructure that includes roads and buildings, power outages, water shortages and disrupted communication, all of which interrupt healthcare delivery and complicate the provision of care [6]. While there is substantial research on the effects of floods on people’s physical health [7, 8], there is a paucity of primary studies examining the disruption they cause to health systems [9–11]. A recent systematic scoping review of published literature on the impacts of flooding on healthcare delivery and health workforce in Australia identified only 13 original research articles and no reviews that provide an overarching framework for understanding the scope of health system impacts [11]. 

Australian medical students on rural clinical placements live in communities for up to 12 months and are embedded within the health system across various settings. This positioning provides rare insights into the breadth of impacts of disaster events on health systems. Medical students’ exposure to clinical practice across diverse healthcare settings, including in disaster contexts, provides a unique opportunity to build understanding of and readiness for broad health system impacts. At the same time, engaging students to reflect on healthcare delivery in disaster affected communities presents an opportunity to achieve educational objectives of integrating social determinants of health concepts into medical education [12, 13]. 

This study draws on the insights gained by medical students placed in diverse clinical settings to explore the ongoing system-wide long-term impacts of flooding on health systems.

### Setting

The Northern Rivers region in northern New South Wales is a highly flood-prone area [14]. On 28 February 2022, the region experienced catastrophic flooding, including an unprecedented 14.4 m high flood in the rural city of Lismore, peaking at 2 m above previous records. This was followed by an 11.4 m flood on 30 March. These catastrophic events caused loss of life and widespread injuries, damaged or destroyed over 10,000 homes, and led to widespread population displacement, and ongoing grief and distress [6, 15, 16]. 

The floods also resulted in severe disruption and damage to healthcare infrastructure [6, 17]. According to the Royal Australian College of General Practitioners, 58 of 70 primary care providers in the region (83%) reported flood damage [18]. Health services were already stretched when the floods occurred, just two months after the COVID-19 epidemic peak in the region.

Medical students come to the region from their universities through the Australian Government’s commitment to build a rural health workforce, with clinical placements facilitated by the University Centre for Rural Health (UCRH) [19]. Placements are provided in various hospital and community settings to meet each university’s curriculum requirements, with a focus on being embedded within rural communities and learning about their healthcare context. Students undertake acute hospital placements in critical care, paediatrics, obstetrics and gynaecology, mental health, medicine and surgery, and in small rural hospitals, community centres, general practices and Aboriginal Medical Services. Previous research with medical students who were on placement in the region during the flooding events showed that they experienced many of the direct, indirect and ongoing impacts of flooding faced by others in the community [20–22]. 

## Methods

### Study design

This study used a qualitative descriptive design, with data collected through focus groups and interviews. Reporting was guided by the Consolidated Criteria for Reporting Qualitative Research Guidelines (COREQ) [23]. Further details of the methods can be found in Supplementary file 1.

### Participants and recruitment

Thirty-nine medical students from three universities, either undertaking or just completing a full-year clinical placement at the UCRH, were invited via email by JB to participate in a focus group. Where students were unable to attend at the scheduled times, they were offered the option of an individual interview.

**Fig. 1:**
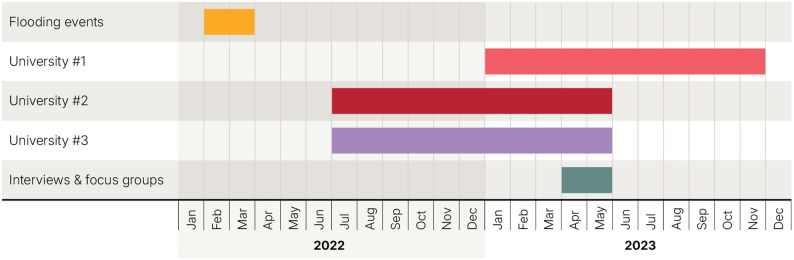
Timeline of flooding events, clinical placements and data collection

JB, RMc, KS and RB had no role in grading or supervising students at the time of the study. They may, however, have been known to participants through their roles as medical and research educators at UCRH. Study materials emphasised that participation was voluntary and would not affect students’ grades, supervision, or relationships with the researchers or the University. They received no compensation for participation. Figure 1 shows the timeline of the flooding events, student placements and data collection.

### Data collection

Data collection (17 April-12 May 2023) commenced 14 months after the initial February 2022 flood. Participants could pause or withdraw at any time and were provided with information about counselling and local support services.

In-person and online focus groups and interviews provided 5.4 h of audio recordings that were professionally transcribed (see Supplementary file 2 for a copy of the data collection tool). Five focus groups were conducted (1 × 10, 1 × 14 and 3 × 2 participants), along with two individual interviews. Participants were initially invited to attend a focus group; however, if they were unable to attend at the scheduled times, they were offered an individual interview. All sessions were facilitated by JB, an experienced qualitative researcher experience (JB), familiar with trauma-informed practice [24]. After each session, JB prepared a reflective summary. Participants were not invited to review transcripts.

### Data analysis

Given the limited literature on health system disruption from flooding, and the lack of a suitable framework for data analysis, we conducted an inductive qualitative content analysis [25]. This approach is consistent with a qualitative descriptive design and allowed categories to be developed directly from the data. The following steps were taken to ensure rigour: (1) Verbatim transcripts were analysed in QSR NVivo V.14.23.3; (2) JB immersed herself in the data by reading and re-reading the transcripts, making reflective notes throughout the process; (3) JB inductively open-coded the data, writing notes and headings to describe the content, which were then used to develop a set of categories; (4) Through comparison and re-reading, JB grouped similar categories into broader sets for review by RB. Consistent with qualitative descriptive approaches, we focused on manifest content and did not quantify the frequency of codes or categories.

As analysis progressed, we observed intersecting impacts across the dataset and required a structured way to organise the findings. We therefore drew on concepts from health system strengthening and system thinking [26, 27], recognising health systems as dynamic, interdependent, and relational. This lens aligned closely with what was emerging in the data.

**Fig. 2:**
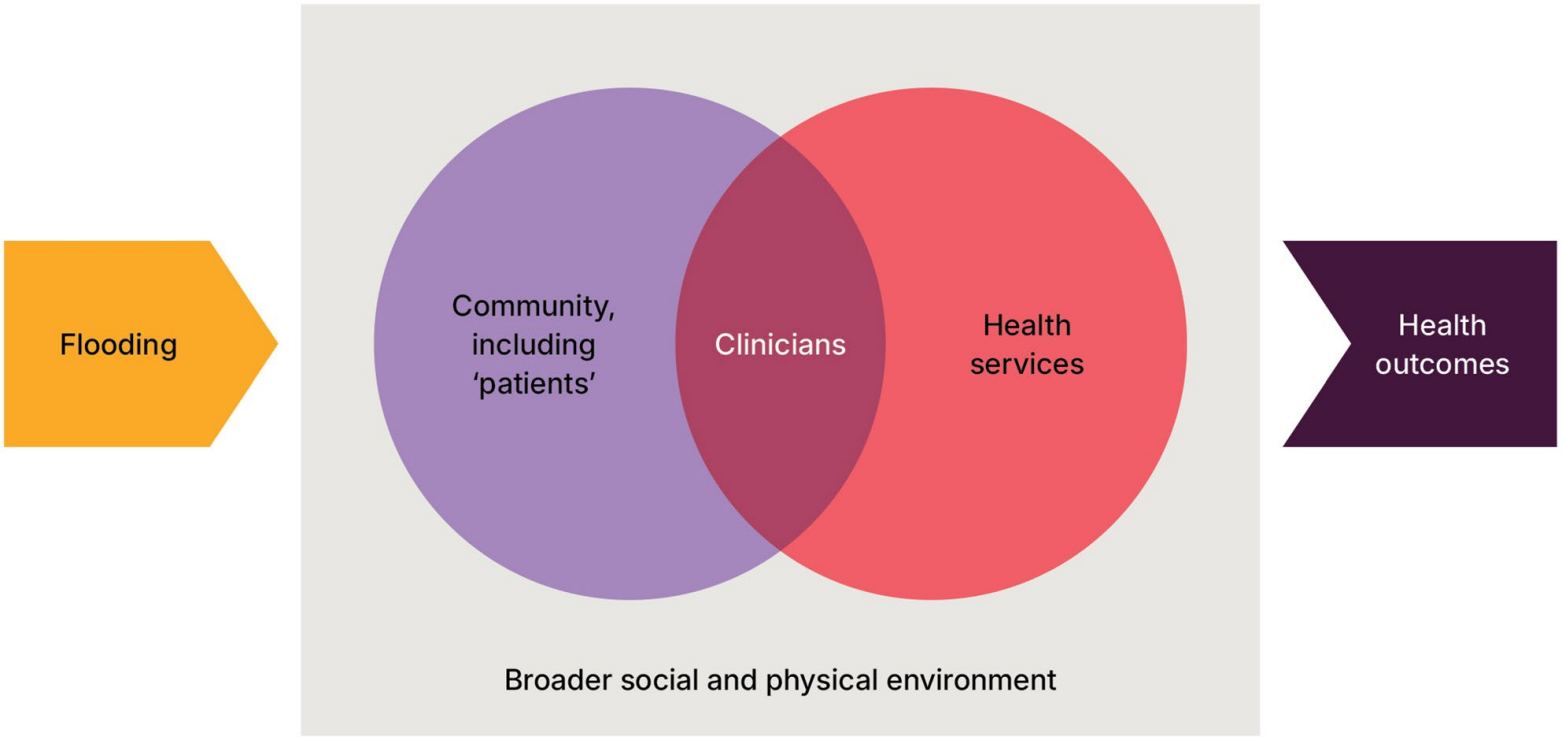
Analytical framework showing the intersecting impacts of flooding on health systems. Notes: The framework emphasises the intersecting impacts of flooding. In this framework, we define ‘community’ as including people who were directly and indirectly impacted by the flood events, including patients who were receiving care by clinicians. ‘Clinician’ includes all health professionals, medical practitioners and health professional students, such as medical students. As members of the community, clinicians are personally affected by the disaster, either directly or indirectly, leading to disruptions in both their personal and professional lives. For this reason, we placed clinicians within the circles of health service and community. ‘Health service’ is intended to be broader than individual impacts on clinicians and includes physical damage to infrastructure and disruptions to delivery of care. We view the ‘broader social and physical environment’ as encapsulating the non-medical factors influencing health outcomes, including the conditions in which people live and work

Using this approach JB and RB developed an analytical framework that describes how floods create intersecting impacts. These impacts affect community members, clinicians (many of whom are also community members), the wider health workforce and health services. All of this occurs within a broader physical and social context (Fig. 2).

We considered data saturation to be reached when no new categories were identified. The initial coding and development of categories were conducted inductively. The analysis and interpretation were then refined through discussion from the wider authorship team, including clinicians who worked in the region during and after the floodings (see Authors’ Note). These discussions focused on clarifying meaning and applied relevance. The process was iterative and involved multiple reflexive discussions over several months.

## Results

### Participant characteristics

Of the 39 medical students invited to participate in the study, 32 (82%) agreed to be part of a focus group or interview. At the time of data collection, most (75%, *n* = 24) had been on placement in the region for 10 months, arriving approximately three to four months after the second flooding disaster. Eight of the students had been in the region for four to five months, arriving approximately 10 months after the second flood event. All students had experienced a variety of clinical placements as described above.

Table 1 provides exemplar quotes to support the findings described in the text below.

**Table 1:** Exemplar quotes to support the findings

System domains	Categories identified	Exemplar Quotes
Community	Community psychological trauma	*“If you went around Lismore…*,* the infrastructure that was damaged is very obvious to see*,* but for the most part*,* people*,* they seem kind of quite normal*,* …but as soon as you go into the clinical setting… there’s clearly a lot of trauma*,* a lot of emotional response that’s just not shown publicly.”* (Focus Group 3) *“There did seem like a lot of mental health presentations to emergency. I’ve had a few doctors talk to us about it and how it (the flood) may be affecting the patients.”* (Interview Number 5) *“Definitely in psych I noticed it a lot*,* people with pre-existing mental health conditions have this thing [flood] that just tips them over the edge and it’s just been this such prolonged trauma.”* (Focus Group 2) *“In anaesthetics*,* we were consulting a patient who had pain for malignancy and*,* during the consult*,* [the] floods came up [and] the patient became visibly distressed… It has permeated through my experience of patients in the hospital*,* but not necessarily directly linked to their diagnoses.”* (Focus Group 6)
Health services	Health service disruptions	*“Their main practice (general practice) was completely devastated*,* and they had to make do with a smaller residence*,* which then meant less clinic rooms. Some staff had left because of the flood and gone back to Queensland or back to metro New South Wales. They had to do the renovations for that property and then they’re also doing the renovations on their main practice*,* so they can’t see as many patients*,* they don’t have as much equipment. They’re also spending all their free time scrambling to get grants and funds to do the best they can with what they’re given.” (Focus Group 1)*
	Staffing challenges	*“Almost every nurse I met in the psych department was a locum after the floods*,* and apparently that wasn’t the case before the floods. I think it just became a much less attractive place to come to as a permanent resident.”* (Focus Group 2)
	Discharge delays	*“A lot of patients come out of hospital with crutches*,* say*,* and they live in a caravan. We can’t actually really discharge them until they have gone to rehab for six weeks*,* and we agree that they can get up their six stairs into the caravan with their total hip replacement crutches. So*,* like*,* normally you could discharge that patient home after a week.”* (Focus Group 1) *“They’re staying in hospital because they can’t get a rehab bed and then they are predisposed to infections.”* (Focus Group 1) *“You can’t discharge them unless they have somewhere to live*,* so trying to find them somewhere to live.”* (Focus Group 7)
Clinicians	Psychological impacts on healthcare workers	*“The GPs … are … trying to really cram about 10 different things into one appointment where they’d normally do two things. They go*,* ‘you actually do need your driver assessment in six months. Let’s do it right now so that you don’t have to come back in six months’. That’s obviously making consults longer*,* which is then making them late. So*,* then they’re finishing later*,* so the GPs’ mental health – you know*,* it’s a whole cycle.” (Focus Group 1)* *“The doctors have empathy fatigue. I think that they have their own trauma through going … the flood and the trauma of having to deal with the patients who have gone through the flood.”* (Focus Group 1)
	Burnout affecting medical education	*“The flood has burnt out [some] doctors. I think it’s underrated*,* the effect that burnt-out doctors have on med students. you’re kind of*,* not resentful*,* but I would say often there’s a space that’s not provided for us’.”* (Focus Group 1) *The burnout … it is compounded by what this community has been through – but it’s like*,* well*,* every community is going to go through this because of climate change. The burden that GPs have to carry their communities through*,* not just medically but socially. There’s not enough money in the world that would [encourage me to take that on] – so it like completely changed my career trajectory.”* (Focus Group 1)
Broader social and physical impacts	Displacement affecting health care access	*“My first rotation was in psychiatry… I remember being struck by the amount of people who were living in caravan parks and temporary flood accommodation.”* (Interview Number 4) *“Often in psych*,* you’d have presentations where they maybe had unstable homes or not necessarily a home address*,* which was a difficulty in discharging them… I don’t know if they lost their home and no longer have an address because of the flood*,* or because the financial hardship they’ve gone through*,* they’re now in a difficult financial situation.” (Focus Group 2)* “*Mum with her four kids and a husband in a caravan*,* with a really*,* very high needs child … I just don’t know how this woman is doing it. They were a bit far out and just the struggle of getting to her child’s medical and physio and all these different appointments.”* (Focus Group 2) *“The surgery to initially remove a mass was delayed because of the floods. I think that wasn’t so much related to the hospital*,* but because of her own situation*,* in Lismore. Having something that may not be as*,* you know*,* immediately life-threatening [is] kind of seen in the days following your house being destroyed [as] not the highest priority for them.”* (Focus Group 7)
	Displacement impacting health outcomes	*“I’ve had a few patients who have actually passed away from not seeking health care – I mean like end-stage renal disease – and they haven’t been able to get to dialysis appointments. Or they’re displaced and they don’t have the means to travel to appointments. … Like babies who were born during the floods and … had no home to go back to and were then placed in*,* I guess*,* campsites with inadequate sewerage. So*,* they’re still getting infections to date*,* which is over a year later*,* like respiratory infections. The children haven’t developed properly; they’re not walking.* *“One patient… really felt that his experience and his displacement – he was living in a motel – had really contributed to his chronic illness deterioration.” (Focus Group 7)* *“We had a few patients that had these atypical infections because of the conditions they were living with as consequence of the floods*,* even five to six months [afterwards]”* (Focus Group 3) *“Some of the patients I’ve seen … I know they don’t interact with family and friends as much*,* largely because they’re not as close anymore as they have all had to move away*,* and there’s been increased rates of drinking alcohol.”* (Focus Group 3) *Probably the saddest thing that I have noticed is that people who have drug and alcohol addictions have either gone into withdrawal and not been adequately helped*,* or due to their trauma – sorry – have found their way back [to their addictions even] when we’ve worked quite hard to get them off those… It’s vastly devastated the community*,* and*,* on top of that*,* I’ve seen a lot of domestic violence spark [up].”* (Focus Group 1) *“I think also there’s a bit of a delay in terms of ability to conduct care confidentially. Going to outreach in the caravan parks in Ballina where a lot of people were living who were displaced*,* there were multiple families… in caravans and cabins.”* (Focus Group 3)
	Greater impact on socially vulnerable groups	*“The floods have disproportionately affected people who were already in positions of disadvantage… people who are of lower socioeconomic status*,* or people who have pre-existing mental health conditions*,* are being hit so hard because it’s just one thing compounding another and they don’t have the means to find alternative accommodation or seek help or have the support networks to fall back on during these times.”* (Focus Group 1)
	Delays in government housing aid increasing stress	“*In consults that I had where people were talking about how they were only just getting offers for their houses. The Government was saying that they were rolling that out*,* but a year or a year and a half later*,* it’s still just kind of trickling to the highest in need*,* so they are feeling quite upset and unsupported by that because they’ve just been kind of in limbo with this property and damaged house*,* there’s nothing that’s been done about it. It just adds to the overall stress*,* having this temporary housing situation and this unresolved one where they don’t know whether they will be offered a buy back. Where they’re going to end up is one thing*,* whether they’re going to stay or move*,* whether they have the financial ability to manage that situation.”* (Focus Group 3)

#### Community

##### Community psychological trauma

More than a year after the floods, infrastructure being repaired and businesses reopening gave an impression of recovery, with students observing that patients and clinicians generally appeared to be coping normally in public interactions. However, within hospitals and medical facilities, students reflected that many patients exhibited substantial trauma and emotional responses that were not so visible in everyday public settings, and many doctors experienced burn out.

The prevalence of trauma from flood-related experiences was particularly noticeable in patients presenting in psychiatric and emergency hospital settings, with many recounting stories of loss, financial hardship and other negative consequences. Other patients were re-traumatised by reference to the flood event, even during interactions about medical conditions or diagnoses that were unrelated to the floods. For instance, one student observed a patient’s distress during an anaesthetic consultation when the floods were mentioned.

This psychological trauma among patients had indirect impacts on clinicians and health services. For example, students highlighted the ongoing challenges faced by some dialysis patients in ensuring consistent access to treatment, particularly in adverse weather conditions when travel safety is uncertain. The necessity of multiple visits to a dialysis centre, for example, poses a significant logistical issue when factors like heavy rain or unsafe driving conditions occur. Patients then need to weigh the risk of being unable to return home against missing their treatment, often opting to stay home to avoid the possibility of being stranded. These stresses and uncertainties for patients in turn causes stress and uncertainty for clinicians through concern about their patients not receiving necessary care.

#### Health services

##### Health service disruption

The flood waters inundated some health facilities, including specialist rooms, general practices, allied health services and pharmacists, making them unsafe for health workers.

Significant damage to healthcare facilities led to closures and relocations, making it more difficult for patients to access care. For example, some specialists whose consulting rooms flooded took earlier-than-anticipated retirement, creating gaps in the workforce and impacting access to services. Similarly, as some general practices were flooded, patients struggled to get appointments with other clinics, even up to a year post-flooding, with the issue being more acute immediately post-floods.

Clinicians found it challenging to stay updated on which health services had reopened or remained closed due to flood damage, with patients sometimes not referred to services due to perceptions of unavailability. Having to keep track of service availability added another layer of complexity to delivering timely healthcare.

Significant delays in accessing psychological care were reported, with individuals requiring psychiatric or psychological support often waiting three to six months. One patient with pre-existing mental health issues experienced further trauma when their house flooded and they had to live in a badly damaged home. In the floods’ initial aftermath, patients benefited from a surge in psychological support but this gradually diminished, highlighting a decline in available assistance when it was still needed.

##### Staffing challenges

With some clinical staff departing from the area post-floods, and difficulties in recruiting individuals for permanent roles in a flood-affected community, students perceived a dependence on locum medical and nursing staff. A lack of accommodation was reported to exacerbate difficulties recruiting staff.

##### Discharge delays

Some patients experienced prolonged hospital stays due to the absence of appropriate housing, particularly for those requiring mobility aids or post-procedure care. This arose because homes had been inundated, leaving them to live in damaged homes or temporary accommodation like caravans, or regular caregivers’ residences were also affected. Students described a patient who was initially hospitalised due to a fall, but could not be discharged because they were living in a caravan in the front yard of their flood-damaged property and could not navigate the caravan steps. The suitability of patients’ homes was also compromised by factors such as damaged driveways, lack of heating, and unstable supply of power and/or clean water. Moreover, rehabilitation facilities were at full capacity post-floods, prolonging hospital stays for many. This increased demand on rehabilitation beds and resources altered the usual flow of post-operative care and delayed the return home of many patients after routine procedures.

#### Clinicians

##### Psychological impact on healthcare workers

Clinicians, as members of the community, were impacted directly and indirectly by the floods. Numerous students perceived doctors, particularly those in general practice, as experiencing ‘burnout’ or ‘empathy fatigue’ due to their flood-related trauma and the continuous strain of treating patients affected by the floods. This situation led to feelings of being overworked. Given the extensive disruptions to healthcare services, doctors responded by prioritising immediate patient needs and striving to deliver comprehensive care. However, this increased consultation times, leading to scheduling delays and uncertainty around follow-up appointments that further impacted doctors’ mental health and workload.

##### Burnout affecting medical education

The floods significantly affected the ability of doctors to mentor and educate students, particularly general practitioners due to increased workloads and perceived burnout. Some students reconsidered pursuing a career in general practice, as they observed how overwhelming the responsibility of supporting communities post-disaster was for some. These experiences underscored the challenges faced by General Practitioners in managing extensive community needs amid climate-related crises.

Despite these difficulties, students gained valuable communication and rapport-building skills from observing interactions between patients and doctors with long-standing relationships. Structured debrief sessions with supervisors were reported to provide valuable opportunities for reflection and meaning making, with students sharing insights into the complex determinants of health and the diverse ways such events impact individuals and communities.

#### Broader social and physical environment

##### Displacement affecting healthcare access

Students observed that some individuals affected by the floods were living in temporary housing such as caravans, with friends and family, or in flood-damaged homes, underscoring the stress of potential relocations and financial instability. During psychiatry rotations, students observed the complex interplay between high numbers of patients living in insecure housing and mental health hospitalisations.

Numerous accounts told of families, including those caring for children with disability, now living in caravans, and struggling with the logistical difficulties of attending multiple medical and social care appointments. One case involved a flood affected family managing life in a caravan for more than 12 months with three children – one of whom had a significant disability. Another highlighted the struggles of a single mother now forced to live in a caravan with her four children, one with high needs, and the immense difficulty of accessing necessary medical and physiotherapy appointments.

Student reports indicated that some people in temporary housing delayed medical treatments, such as surgery for non-immediately life-threatening conditions. Disrupted living situations meant that resolving immediate housing needs often took precedence over healthcare concerns.

Other students noted significant challenges in organising follow-up health and social services due to patients’ housing displacement. During a general practice placement, one patient highlighted this issue by saying, *“It’s difficult to organise home visits or care nurses at home when there is no home.”* This sentiment was echoed by another when discussing potential services outside the practice and hospital. The patient, having lost her home in the floods, pointed out the irony in the term “home care” when their displacement rendered such services nearly impossible. This underscores the broader impact of housing instability on accessing essential health and social support.

##### Displacement impacting health outcomes

Students described the potentially dire health consequences of community members not being able to access healthcare because of living in temporary housing in the aftermath of the floods. These included cases where patients with end-stage renal disease could not access dialysis appointments, due to being displaced far from their home base and/or lacking the means to travel, which sometimes resulted in fatalities. Additionally, some babies born during the floods were placed in inadequate living conditions, such as campsites with poor sewerage systems, leading to ongoing infections up to a year later.

Students reported that the pervasive influence of the floods affected patients’ experiences in the hospital, even when the floods were not directly connected to their medical conditions or diagnoses. Some attributed their worsening chronic illness to stress and uncertainty due to being displaced from their homes, even several weeks after the flooding events, highlighting the disaster’s ongoing repercussions on individuals’ health.

Students observed patients presenting with atypical infections, some persisting several months post- floods, that were perceived to result from poor living conditions following the disaster and cleaning flood-damaged properties. Reports emerged of patients presenting with scabies, a condition that is seldom encountered by students and was linked to the compromised living conditions of the temporary housing allocated to flood victims. The close proximity to others and lack of adequate washing facilities were key factors in the spread of the condition.

Residents in severely impacted communities have been unable to return home since the disaster, resulting in adverse effects on social cohesion and health. Some students referred to these residents as “climate refugees,” highlighting that this term, often associated with international contexts, is also relevant domestically. Students observed a rise in social isolation among people displaced from the community, leading to increased alcohol consumption and domestic violence.

There were also challenges in providing confidential care in temporary living situations, such as caravan parks, where many displaced families resided. Healthcare outreach efforts in these settings faced difficulties due to lack of privacy, with multiple families or individuals sharing close quarters and potentially overhearing conversations about personal or sensitive issues.

##### Disproportionate impact on socially vulnerable groups

Students observed that the floods significantly worsened the circumstances for local Aboriginal communities and individuals already grappling with socioeconomic challenges and pre-existing mental health issues. The heightened vulnerability of disadvantaged groups, who lack resources, alternative housing options and robust support networks in flood-affected areas, led to severe and prolonged disruptions to their health and wellbeing compared to other populations.

##### Delays in government housing support increasing stress

Delays in government accommodation assistance and unresolved housing situations, including prolonged wait times for government offers on housing repairs or buybacks, further increased stress and uncertainty for affected individuals.

## Discussion

This study offers new insights into the sustained, multifaceted, and intersecting impacts of a major flooding event on health systems in a rural Australian context. Drawing on the experiences of medical students and the perspectives of clinicians based in the region during and after the disaster, it presents an analytical framework to capture these dynamics. The findings highlight four interrelated domains of impact - community, health services, clinicians, and broader social and physical environments. Across these domains, ongoing psychological distress within communities, prolonged disruption to health service delivery, and persistent staffing and discharge pressures were evident well beyond the immediate response phase. The emotional toll on healthcare workers was significant, with burnout continuing to affect clinical care and the quality of medical education. Displacement due to flooding created barriers to accessing care and contributed to poorer health outcomes, particularly for socially vulnerable groups. Delays in housing support further compounded stress and instability in already impacted populations.

An overlooked aspect of healthcare disruption during weather-related disasters is the impact on clinicians, who may be personally impacted by the disaster, further exacerbating disruptions to healthcare services. Students in our study reported that clinicians were often overworked and under immense stress, resulting in physical and emotional exhaustion, and references to ‘empathy fatigue’. Such conditions can compromise their ability to deliver quality care, negatively impacting patient outcomes. Students reported that clinician ‘empathy fatigue’ and burnout also impacted effective supervision. The observed strain on general practitioners also influenced some students’ career intentions, steering them away from general practice. Consistent with the findings of the review by Zurynski et al. [4] and Dorfer et al. [11] we found that the flooding events had a deleterious effect on the broader health workforce, which included psychological impacts, unsafe working conditions, absenteeism and health system breakdown. Alzailai et al.’s [28] review of effects on healthcare staff working in disasters identified a need for interventions to reduce the risk of burnout and maintain quality of patient care. The lack of other research examining the impacts of disasters on clinicians’ ability to supervise students highlights the need for research to understand how such events affect clinical education and to develop strategies to support clinicians and students during and after disasters.

While the data from this study refer to impacts of flooding on some health outcomes, the focus of our study is primarily on the impacts on health systems. The concern is, of course, that disruption to health systems will have flow on effects to health outcomes. The rationale for our focus on health systems is that enhancing understanding of the relationships should allow for more tailored responses for the purpose of minimising impacts on health outcomes. Furthermore, understanding impacts on systems is especially important to protecting the health of socially vulnerable groups that typically are more reliant on well-functioning systems as they often have fewer resources to draw on in times of crisis.

Displacement from homes due to flooding was reported by students as a major stressor and cause of ill-health across the Northern Rivers region. Evidence from the 2017 flood in this region identifies that being displaced for more than six months is a major risk to mental health [29, 30], a finding consistent with international reviews documenting the health impacts of displacement as a result of climate change [31, 32]. Inadequate housing for residents and health care workers was identified, with housing insecurity amplifying psychological distress and undermining the health system’s capacity to respond effectively. More broadly, the psychological effects of flooding were evident across all categories, even when mental health was not the primary focus of discussion. These findings indicate that timely, accessible, and sustained psychosocial support is essential - not only in the short-to-medium term but also in the longer term - as recovery and adaptation extend over years. The need for ongoing psychological support is particularly acute where housing insecurity remains unresolved, as prolonged instability in living conditions compounds distress and delays recovery. Such support should be regarded as complementary to, rather than in competition with, the urgent need to restore the physical environment. Collectively, they highlight the necessity of sustained government and institutional investment in both psychosocial support and housing security as integral components of disaster recovery and preparedness.

Our previous research in the Northern Rivers showed that medical students who were in the region during the 2022 floods experienced considerable stress and disruption to their learning [20, 22]. The students in the study reported here were not in the region when the floods occurred, but they experienced the ongoing health system impacts through their clinical placements and interactions with communities.

The depth and range of reflections from medical students on the impacts of flooding and their determinants were a striking feature of our research. Some students had considered these issues beforehand and discussed them during debrief and reflection groups with a clinical mentor at the UCRH during their placement. It was also apparent that the focus group discussions enabled the students to process and reflect on what they had learned from their clinical placement experiences. Although social determinants of health education for medical students is deemed necessary, the optimal teaching and evaluation methods remain unclear. A review article on medical education regarding social determinants of health reports the most common approach is experiential learning through community-based learning [33]. In our study, we found that students’ various community placements facilitated this experiential learning. Additionally, the process of critical reflection, whether through debriefing and reflection sessions or discussions as part of this study, enabled students to recognise the diverse impacts of flooding. While critical reflection is valuable for transforming learner attitudes, few studies have examined students’ reflexivity. Given that rural placement curricula often focus on community-based medicine, there is a strong opportunity to harmonise social determinants of health education for medical students. Medical students represent the future medical workforce who will be required to practice in the context of a changing climate, making it vital that they are equipped with the knowledge, skills, and resilience needed to respond effectively [34, 35]. As climate change increasingly amplifies inequities across social determinants of health, further research is needed to identify the most effective approaches for teaching medical students about these determinants so they can integrate this understanding into clinical practice.

### Limitations

Limitations include the context specific nature of some flood impacts, the relatively small student cohort, and the students’ limited experience of working in the health system. However, the insights provided resonate with our clinical coauthors who have many years of experience working in primary care and hospitals in the region prior to, during and after the floods (CA and JD). JB, CA, JD, RMcN and RB are embedded researchers at the UCRH in Lismore, which was at the epicentre of the flooding events and may have influenced both their approach to the study and their perspectives on the data.

## Conclusion

Our study draws attention to the multifaceted, interlinked impacts and long-lasting impacts of natural disasters such as floods on health systems, including through the impacts on a health workforce who are part of the local community, on healthcare delivery and through social determinants of health. As the climate warms, the increase in catastrophic weather events will accelerate their impact on health systems. More evidence and appropriate policy are needed on how best to prepare health systems to withstand these complex events. Our findings also show that placements in disaster-affected settings can provide valuable experiential learning opportunities, though these were maximised when accompanied with structured opportunities for reflection that help students consider social determinants and differential impacts. Medical students are the next generation of the health workforce, and it is essential that they are adequately prepared for practising in a changing climate. We anticipate that the analytical framework developed for this study may be useful when undertaking literature reviews, designing and implementing empirical research on the impacts of climate-related disasters on the health system, and guiding interventions to ameliorate these impacts.

## Supplementary Information

Below is the link to the electronic supplementary material.

Supplementary Material 1

## Data Availability

The raw datasets generated and analysed during the current study are not publicly available due to the fact that ethical approval and consent was not obtained for data sharing publicly but are available from the corresponding author on reasonable request.

## Declarations

### Authors information

JB is a Senior Research Fellow at the University Centre for Rural Health (UCRH) and leads the evaluation of its medical education program. KS is a Professor of Medical Education at the University of Sydney and plays a key role in evaluating the broader medical program. CA is a General Practitioner and Clinical Educator at the UCRH, involved in teaching medical students. JD is a Specialist Respiratory Physician at Lismore Base Hospital and a Clinical Educator at the UCRH, also supporting medical student education. RMc is a Research Fellow at the UCRH with expertise in climate change adaptation. RB is an Honorary Professor of Rural Health at the Sydney School of Public Health, University of Sydney, based at the UCRH, and leads research on the health impacts of climate change. JB, RMcN, CA, JD, and RB are all based in Lismore, where the flooding events occurred. KS is based in Sydney and works externally to the day-to-day operations of the UCRH.

## Abbreviations

SDOH: Social determinants of health
UCRH: University Centre for Rural Health
