# Supplementary material for: Longer-term impacts of flooding on Australian health systems: insights from medical students on rural clinical placement
Source: BMC Health Serv Res. 2026 Feb 19;26:401. doi: 10.1186/s12913-026-14171-4 (PMC13019839; doi:10.1186/s12913-026-14171-4)
Supplement: Supplementary file 1 — Supplementary Material 1 [file 12913_2026_14171_MOESM1_ESM.docx]

**Supplementary file 1**

*Table 1: Additional information as per the consolidated criteria for reporting qualitative research (COREQ) checklist*

| **Domain 1: Research team and reflexivity** | |
| --- | --- |
| **Personal characteristics**   1. Interviewer/facilitator; 2. Credentials; 3. Occupation; 4. Gender; 5. Experience and training | Data collection was conducted by JB, a qualitative researcher with extensive experience in facilitating focus groups and interviews, and working within trauma-informed practice.  All authors are employed at academic institutions: JB, RMc and RB are in research-focused roles; KS, CA, JD hold medical education-focused roles. JB, CA, JD, RMc, RB are also employed and/or based at the UCRH in the Northern Rivers; KS is employed by the University of Sydney and based in Sydney; KS and RB hold professorial roles.  JB, RMc, CA, JD and RB were residing in the community at the time of the flood and, although not directly affected, witnessed both the immediate and ongoing impacts on the community and on the region’s health services.  RB and JD are male, and JB, CA and RMc are female. |
| **Relationship with participants**  6. Relationships established; 7. Participant knowledge of the interviewer; 8. Interviewer characteristics | JB is Senior Research Fellow at the UCRH, employed in the evaluation of the medical and allied health program and in rural health research. JB, RMc KS, and the senior author (RB) did not have a role in grading or supervising the students at the time of the study.  The researchers may have been known to the participants through their roles as medical and/or research educators at UCRH.  The email invitation, consent form, and participant information sheet clearly stated that participation was voluntary and that choosing not to participate - or to withdraw - would not affect students’ grades, supervision, or any current or future relationship with the researchers or the University of Sydney. |
| **Doman 2: Study design** | |
| **Theoretical framework**  9. Methodological orientation and theory | The team used a qualitative descriptive design. |
| **Participant selection**  10. Sampling; 11. Method of approach; 12; Sample Size; 13. Non-participation | Purposive sampling techniques were used to ensure a diversity of views. Thirty-nine medical students undertaking a rural clinical placement at the UCRH at the time of the study were invited via email by JB to participate. To follow up on those who did not respond to the first invite, another email was sent.  In total, seven of the 39 students did not participate. While no one explicitly declined to be involved, we were unable to find a suitable time for some to participate. There was no compensation offered for being part of the study. |
| **Setting**  14. Setting of data collection; 15. Presence of non-participants; 16. Description of sample | The setting is the Northern Rivers region of New South Wales, Australia up to 14 months following catastrophic flooding events in February and March 2022. The medical students involved in the study were undertaking a rural placement at the University Centre for Rural Health in Lismore. The students were placed in the region after the flood event, with the majority being in region for 12 months, approximately four to five months post second flood in 2022. Students come to the region as part of the Australian Government’s commitment to build a rural health workforce. The students were all in the third year of their course and were from three different Australian universities.  Only the researcher (JB) and students were present during data collection. |
| **Data collection**  17. Interview guide; 18. Repeat interviews; 19. Audio/visual recording; 20. Field notes; 21. Duration; 22. Data saturation; 23. Transcripts returned | The focus group/interview guide was developed by authors JB, KS and RB. There were no repeat interviews.  All interviews were audio recorded, professionally transcribed and checked for accuracy. Transcripts produced did not distinguish individual speakers.  Reflective memos were generated by the interviewer immediately after completion of the interview or focus group.  Interviews and focus groups provided 5.4 hours of audio recordings.  As part of our ethics procedures, a distress protocol was implemented. Participants were reminded that they could pause or withdraw at any time without consequence. If distress had been observed, the discussion would have been paused and support offered, and participants were provided with information about university counselling and local support services.  Given our defined cohort of medical students, data saturation was considered reached during analysis when no new categories emerged, with later data contributing only depth to existing categories.  The participants were not offered an opportunity to review the transcripts. |
| **Domain 3. Analysis and findings** | |
| **Data analysis**  24. Number of data coders; 25. Description of the coding tree; 26. Derivation of themes; 27. Software; 28. Participant checking | Guided by Elo and Kyngas the following steps were undertaken: 1) Verbatim transcripts and reflective summaries were uploaded into QSR NVivo V.14.23.3. 2) JB immersed herself in the data by reading and rereading the transcripts and making reflective notes. 3) She inductively open-coded the data, writing notes and headings to describe the content, which she then used to develop a set of categories. 4) Through comparison and rereading, JB was able to group similar categories into broader sets for review by RB. 5) Based on this analysis of the data and drawing on health systems and system thinking literature, JB and RB developed an analytical framework for describing how, at a local or regional level, floods have intersecting impacts on members of the community; individual clinicians (many of whom are members of the community); the broader health workforce, and health service; with all impacts occurring in the broader physical and social context. 6) The analysis, interpretation and framework development were refined through input from the other authors.  Consistent with qualitative descriptive approaches, we focused on manifest content and did not quantify the frequency of codes or categories. |
| **Reporting**  29. Quotations presented; 30. Data and findings consistent; 31. Clarity of major themes; 32. Clarity of minor themes | We report our results using quotes that are illustrative of categories. Because speakers could not be reliably re-identified across the recordings, quotations are reported at the focus-group level. Consistency between data and findings were iteratively checked throughout the analysis to ensure consistency and accuracy between the two. |

**Supplementary file 2**

The questions below are intended to be a guide.

1. Why did you choose this rural placement?

*Knowledge of disaster prior to placement*

1. Did you know before you came to the area there had been a flood? What did you know about this, and where did you get your information from?
2. Was this a factor in your decision making to come to the Northern Rivers?

*Observations – mental and physical impacts and vulnerable populations*

1. Tell me about your experience of placement in a flood impacted community.
2. What did you observe whilst on placement regarding the impact of the flood on patients (prompts: mental and physical health and wellbeing, displacement, compounding trauma related to bushfires, COVID and floods)
3. Were their groups that were more vulnerable to impacts? (Prompts: Aboriginal people, people with disability, carers, LQBTIQ, low socioeconomic, children) How did this look in practice?
4. How was health care delivery impacted (prompts observations and experiences as a student, access barriers to services)
5. Are there things that stood out for you? What were the stories you heard or experienced that really stood out for you?

*Individual development and adaptability*

1. While on placement were their things that you needed to do differently than in previous placements or in ‘a normal situation’ ? If so, what was different? (Prompts: interactions with patients/carers and hospital/community health staff, considerations of psychosocial impacts on health, clinical reasoning, procedures)

- How did you deal with these differences?
- Did you come up with new activities or solutions yourself?

1. Who or what was helpful when performing things differently?
2. Who or what was hindering when performing things differently?
3. Were there any times when you were surprised by your own activities?

*Supervision and support*

1. How did your existing knowledge and skills add value to what you were able to contribute. What types of skills and knowledge may have equipped you further? (Prompts: trauma informed care approaches, knowledge of specific vulnerable groups).
2. What was your supervision arrangements and was your supervision impacted? If so, how? – If yes, do you think this was related to your supervisors’ experience of the flood events / experience providing care to this community?’
3. Did you feel adequately prepared to talk with and support flood affected people? Were you left feeling that you had served a purpose on your placement, or did you feel disempowered?
4. How did you manage your feelings? Did you engage in any self-care? If so, what kind?
5. Did you seek help from peers, supervisors, or seniors? If not, who could you have approached for information and advice? Was it a safe environment for discussions?
6. Looking back, what type of support and preparedness would have been helpful for you? Would any form of medical school training been helpful?

*Career intentions and comparison to other placements*

1. How has your experience compared with other placements you have had? What have you learnt from it?
2. Has your experience changed your career intentions in any way? If so, how?

*In conclusion*

1. Is there anything I didn’t ask about that you would like to add?
